# Survival outcomes and prognostic factors of lung cancer patients with the *MET* exon 14 skipping mutation: A single-center real-world study

**DOI:** 10.3389/fonc.2023.1113696

**Published:** 2023-03-09

**Authors:** Chien-Hung Gow, Min-Shu Hsieh, Yi-Lin Chen, Yi-Nan Liu, Shang-Gin Wu, Jin-Yuan Shih

**Affiliations:** ^1^ Department of Internal Medicine, Far Eastern Memorial Hospital, New Taipei City, Taiwan; ^2^ Department of Internal Medicine, National Taiwan University Hospital and College of Medicine, National Taiwan University, Taipei, Taiwan; ^3^ Department of Healthcare Information and Management, Ming-Chuan University, Taoyuan, Taiwan; ^4^ Department of Pathology, National Taiwan University Hospital, Taipei, Taiwan; ^5^ Department of Internal Medicine, National Taiwan University Cancer Center, National Taiwan University, Taipei, Taiwan; ^6^ Graduate Institute of Clinical Medicine, National Taiwan University, Taipei, Taiwan

**Keywords:** adenocarcinoma, immunohistochemistry, *MET* exon 14 skipping, pulmonary sarcomatoid carcinoma, overall survival

## Abstract

**Introduction:**

The *MET* exon 14 skipping *(MET*ex14) mutation is an important oncogenic driver in lung cancer. We performed a retrospective analysis of clinical data from lung cancer patients with the *MET*ex14 mutation to analyze their survival outcomes and associated prognostic factors.

**Methods:**

A one-step reverse transcription-polymerase chain reaction to examine the presence of the *MET*ex14 mutation was performed using RNA samples from 1374 lung cancer patients with no detected *EGFR* and *ALK* mutations. Pathological features and immunohistochemistry (IHC) results for c-MET were analyzed in patients with *MET*ex14-positive tumors.

**Results:**

*MET*ex14 was identified in 69 patients with lung cancer, including 53 adenocarcinoma (ADC) and 16 non-ADC patients. In comparison with patients without the *MET*ex14 mutation, lung cancer patients harboring the *MET*ex14 mutation were generally elderly individuals, never-smokers, and had poor performance scores. A higher frequency of *MET*ex14 mutations was detected in pulmonary sarcomatoid carcinoma (PSC) patients (24.3%, n = 9/37). However, stage IV PSC patients with or without the *MET*ex14 mutations showed similarly poor overall survival (OS) (*p* = 0.429). For all 36 *MET*ex14-positive lung ADCs, multivariate analysis showed several poor prognostic factors, including strong c-MET IHC staining (*p* = 0.006), initial brain metastasis (*p* = 0.005), and administration of only supportive care (*p* < 0.001). After excluding seven patients who received only supportive care, we further analyzed 29 stage IV lung ADC patients with *MET*ex14 mutations who received anti-cancer treatment. Multivariate analysis showed that pemetrexed treatment (*p* = 0.003), lung radiotherapy (*p* = 0.020), initial brain metastasis (*p* = 0.005), and strong c-MET IHC staining (*p* = 0.012) were independent prognostic factors for OS in these patients.

**Conclusions:**

A higher frequency of *MET*ex14 mutations was detected in PSC patients. Stage IV PSC patients with or without the *METex14* mutations had similarly poor overall survival. Pemetrexed-based chemotherapy, strong c-MET ICH staining, initial brain metastasis, and lung radiotherapy, may help predict survival outcomes in patients with advanced lung ADCs harboring the *MET*ex14 mutation.

## 1 Introduction

Acquired gene alterations in lung tumors serve as driver mutations that initiate tumorigenic and invasive abilities. Some of these mutations can be targeted by specific small-molecule inhibitors or monoclonal antibodies (1). The c-mesenchymal-epithelial transition protooncogene (*MET*) is an important gene that encodes the MET protein, which functions as a transmembrane receptor tyrosine kinase and may trigger tumor growth under aberrant activation (2). *MET* exon 14 skipping (*MET*ex14) is one of the most common gene alterations of *MET*, and it acts as an important oncogenic driver in lung cancer (3). The *MET*ex14 mutation results in the loss of the juxtamembrane domain of the MET protein, which regulates and prevents MET over-signaling (4). Consequently, the E3 ubiquitin ligase c-cbl fails to bind to the MET protein, reducing receptor degradation and causing overactivation of MET-mediated signaling, thereby driving oncogenesis (5).

Among patients with lung cancer, the *MET*ex14 mutation occurs in 2%-4% of those with adenocarcinomas (ADC), 1%-2% of those with squamous cell carcinoma, and 7% to 31% of the patients with pulmonary sarcomatoid carcinoma (PSC) (6–8). Several small molecules targeting and inhibiting MET tyrosine kinase have been evaluated for their efficacy in the treatment of *MET*ex14-positive non-small cell lung cancer (NSCLC). Clinical studies have demonstrated that crizotinib, a multikinase inhibitor of receptor tyrosine kinases (RTKs), reduces the tumor size in advanced NSCLC patients carrying the *MET*ex14 mutation (9). However, the phase II METROS study reported limited benefits in terms of objective response rate (ORR), progression-free survival (PFS), and overall survival (OS) (10). Capmatinib, an oral adenosine triphosphate (ATP)-competitive MET inhibitor, demonstrated anti-cancer efficacy with an ORR of 68% and a median PFS of 9.69 months in treatment-naïve patients in the phase II GEOMETRY mono-1 trial (11). Another ATP-competitive MET inhibitor, tepotinib, showed a favorable overall response rate and rapid as well as durable response in the phase II VISION study (12). Thus, both capmatinib and tepotinib are recommended as first-line treatments of choice for advanced NSCLC with *MET*ex14-positive tumors (13). Other MET-specific tyrosine kinase inhibitors (TKIs), multikinase inhibitors, and anti-MET antibodies are currently in ongoing clinical trials for the treatment of this patient population (14).

NSCLC patients carrying *MET*ex14 mutations receive conventional treatments without specific anti-MET therapy and have a poor prognosis and short OS (8, 15). Their OS is comparable to that of patients with undetected major driver mutations (16). Although *MET*ex14-positive NSCLC patients treated with selective MET TKIs reported longer OS, up to 30%-40% of these patients were reported to be non-responders (11, 17, 18). The factors associated with a poor prognosis in these patients remain unclear. Our previous study demonstrated that stage IV patients with *MET*ex14 mutations had diverse survival outcomes; some patients showed very poor survival, while others had a relatively long survival period (16). Therefore, identification of the potential factors that predict OS in these patients is important. In the present study, we performed a retrospective evaluation of clinical data from lung cancer patients with the *MET*ex14 mutation to analyze their survival outcomes and associated prognostic factors.

## 2 Patients and methods

### 2.1 Ethics statement

This study was approved by the institutional review board of National Taiwan University Hospital (NTUH), Taipei, Taiwan. Written informed consent was obtained from all patients before tumor specimen collection for clinical data acquisition and molecular analyses.

### 2.2 Patients

We retrospectively included patients diagnosed with lung cancer at the National Taiwan University Hospital between January 2006 and August 2020. Tumor specimens were consecutively and prospectively collected from either the primary lung tumors or distant metastatic sites by surgery, core needle biopsy, bronchial washing, endobronchial biopsy, and cell blocks of malignant pleural effusion. Only patients with lung cancer with no detected *EGFR* and *ALK* mutations were included in this study. Tumors were confirmed by mutational analysis to exclude co-major driver mutations.

### 2.3 Mutational studies

*EGFR* mutation tests were performed using a one-step reverse transcription-polymerase chain reaction (RT-PCR) with RNA samples. *ALK* mutations were detected by either RT-PCR or immunohistochemistry (IHC) staining using the Ventana ALK (D5F3) antibody. Patients with lung cancer with no detected *EGFR* and *ALK* mutations were examined for the *MET*ex14 mutation. The presence of other major driver mutations, including *KRAS*, *HER2*, *BRAF* V600E, *ROS-1*, and *RET*, was also analyzed. Tumor specimen preparation, RNA extraction, primer selection, RT-PCR conditions, and sequencing methods for all driver mutations were performed using methods described previously (16, 19). Some of the patients with *ROS1* fusion and *RET* fusion underwent fluorescence *in situ* hybridization with a previously described standard protocol (19).

### 2.4 Acquisition of clinical and pathologic data

Demographic characteristics and clinical features of all enrolled patients were obtained from medical records. Patients who smoked less than 100 cigarettes in their lifetime were defined as nonsmokers. The Eastern Cooperative Oncology Group (ECOG) performance score (PS) was used to rank performance status (20). Distant metastases were evaluated and the number of different metastatic sites was recorded. Treatment modalities, including therapeutic surgery, chemotherapy, immunotherapy, MET TKI treatment, and local radiotherapy (RT) at the primary or metastatic sites were recorded. The endpoint of clinical analyses was OS, defined as the time from the initial diagnosis of lung cancer to death or the date of censoring at the last follow-up or loss of contact on April 30, 2022.

### 2.5 c-MET immunohistochemistry staining

MET protein expression was evaluated by performing c-MET IHC staining on formalin-fixed paraffin-embedded (FFPE) tissue sections of *MET*ex14-positive tumors. As described previously, 4-μm-thick FFPE sections were dewaxed, rehydrated, and reacted with a 1:50 dilution of anti-human c-MET antibody clone SP44 (Abcam, Cambridge, UK) (16). Staining was performed using an automated stainer (Ventana Benchmark; Roche Ventana, Tucson, AZ, USA) in accordance with the manufacturer’s instructions. The intensity of MET expression was scored and classified as strong (score 3+), moderate (score 2+), weak (score 1+), or absent (score 0), as described previously (16). Staining distribution patterns were recorded as diffuse, focal, or negative. Other IHC stains, including pancytokeratin (CK), thyroid transcription factor-1 (TTF-1), and vimentin, were assessed as described previously (16). A portion of the IHC data was retrieved from the medical records.

### 2.6 Statistical analysis

Categorical variables were compared using the chi-squared test or Fisher’s exact test when the expected number was less than 5. Continuous variables were expressed as median values with upper and lower values. OS and univariate analyses were estimated using the Kaplan–Meier method and the log-rank test to measure all differences in survival curves. We used a Cox proportional hazard regression model for multivariate analysis of OS with the backward-stepwise method. All tests were two-sided, and differences were considered significant when *p* < 0.05. Analyses were performed using the IBM SPSS software for Windows (version 26.0, IBM Corp., Armonk, NY, USA).

## 3 Results

### 3.1 Clinical features of lung cancer patients with and without the *MET*ex14 mutation

**Figure 1:**
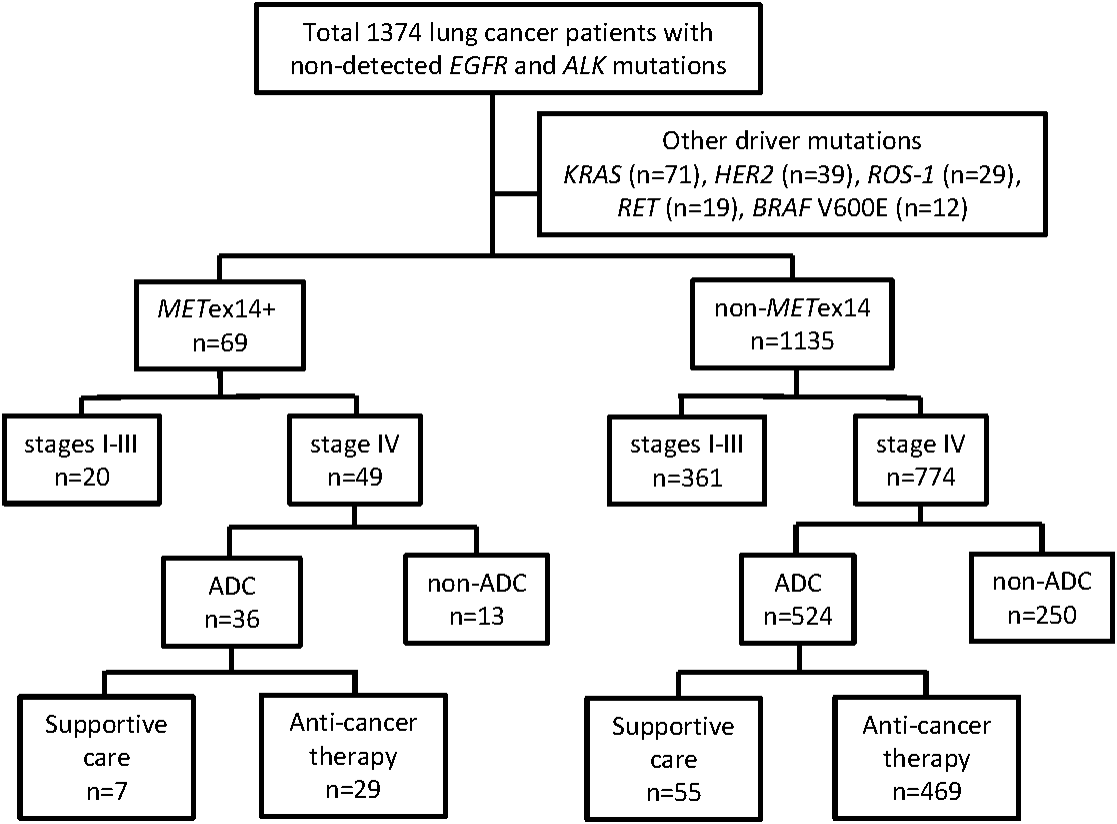
Overview of patient selection and patient groups. ADC, adenocarcinoma.

This cohort study enrolled 1374 lung cancer patients with no detected *EGFR* and *ALK* mutations (**Figure 1**). Among these patients, 170 had other driver mutations were excluded, including 71 with *KRAS* mutations, 39 with *HER2* mutations, 29 with *ROS-1* fusions, 19 with *RET* fusions, and 12 with *BRAF* V600E mutations (**Figure 1**). The *MET*ex14 mutation was identified in 69 patients, including 53 patients with ADC and 16 patients with non-ADC. Some patients with the *MET*ex14 mutation have been described in our previous report (16). In total, 1135 patients who did not show any driver mutations (*MET*ex14, *EGFR*, *ALK*, *KRAS*, *HER2*, *BRAF* V600E, *ROS-1*, or *RET*) were categorized into the non-*MET*ex14 group. Among the 69 patients with *MET*ex14-positive lung cancer, the median age was 74.2 years at initial diagnosis, 44 patients were male (64%), and more than half were never-smokers (41/69; 59%). The majority of the patients (48/69, 69%) had good ECOG PS scores (0-1), while 15 patients (22%) had a PS score of 2 and 6 patients had poor PS scores (3 and 4). A vast majority of the patients had stage IV disease (49/69, 71%). In comparison with the non-*MET*ex14 group, lung cancer patients harboring the *MET*ex14 mutation were generally elderly individuals (≥70 years old, *p* = 0.009), never-smokers (*p* = 0.020), had poor ECOG PS (*p* = 0.026), and showed different subtypes of non-ADC (*p* < 0.001; **Table 1**). The highest frequency of *MET*ex14 mutations was observed in PSCs (9/37, 24.3%), followed by ADCs (53/803, 6.6%), pleomorphic carcinomas (1/19, 5%), squamous cell carcinoma (4/107, 3.7%), NSCLC-not otherwise specified (NOS) (1/80, 1.3%), and small cell lung cancer (1/159, 0.6%; **Supplementary Table 1**).

**Table 1:** Clinical characteristics of lung cancer patients harboring tumors with (*n* = 69) and without (*n* = 1135) the *MET*ex14 mutation.

Clinical characteristic	*MET*ex14+	No *MET*ex14	*p*-value^#^
Patients, n	69	1135	
Age, years			
Median (range)	74.2 (36-95)	67.2 (18-99)	0.013^§^
≥65, *n* (%)	54 (78)	679 (60)	0.002
≥70, n (%)	43 (62)	525 (46)	0.009
Sex, *n* (%)			0.938
M	44 (64)	729 (64)	
F	25 (36)	406 (36)	
Smoking status, *n* (%)			0.020
Current/Ever	28 (41)	624 (55)	
Never	41 (59)	511 (45)	
ECOG PS, *n* (%)			0.026
0−1	48 (69)	929 (83)	
2	15 (22)	130 (11)	
3−4	6 (9)	76 (6)	
Stage, *n* (%)			0.225
I	11 (16)	119 (10)	
II	3 (4)	53 (5)	
III	6 (9)	189 (17)	
IV	49 (71)	774 (68)	
Histology			0.066
Adenocarcinoma	53 (77)	750 (66)	
Non-adenocarcinoma	16 (23)	385 (34)	
Subtype of non-adenocarcinoma			< 0.001
Squamous cell carcinoma	4 (25)	103 (27)	
Sarcomatoid	9 (57)	28 (7)	
Small cell	1 (6)	158 (41)	
Pleomorphic carcinoma	1 (6)	18 (5)	
NSCLC-NOS	1 (6)	79 (20)	

ECOG PS, Eastern Cooperative Oncology Group performance status; F, female; M, male; n, number; NOS, not otherwise specified; NSCLC, non-small cell lung cancer.

#p-values were calculated using the chi-squared test or Fisher’s exact test when the expected number was less than 5.

§Using Kruskal–Wallis test.

### 3.2 Univariate and multivariate analyses of prognostic factors for overall survival in all stage IV Adenocarcinoma patients harboring the *MET*ex14 mutation

We further focused on patients with lung cancer who were initially diagnosed with stage IV ADC, which included 36 patients harboring the *MET*ex14 mutation and 524 patients without major driver mutations (i.e., the non-*MET*ex14 group consisting of patients without detected *MET*ex14, *EGFR*, *ALK*, *KRAS*, *HER2*, *BRAF* V600E, *ROS-1*, and *RET mutations*) (**Figure 1**). We examined the prognostic role of various factors for OS, including age, sex, smoking status, pathologic features of c-MET IHC, ECOG PS, presence and number of distant metastatic sites, and provision of anti-cancer therapy or only supportive care. Univariate analyses of OS was performed using Kaplan–Meier survival analysis in 36 stage IV ADC patients, of which c-MET IHC analysis data were available for 33 patients (**Table 2**).

**Table 2:** Univariate and multivariate analyses of prognostic factors for overall survival in all patients with *MET*ex14-positive stage IV adenocarcinoma (n = 36).

Factor	Patient, n	Univariate analysis^#^		Multivariate analysis^$^	
		Median OS (months)	*p*-value	HR (95% CI)	*p*-value
Age, years					
<70	13	7.8	0.559		
≥70	23	2.5			
Sex					
M	21	3.8	0.298		
F	15	13.6			
Smoking status					
Non-smoker	23	13.6	0.244		
Smoker/ex-smoker	13	3.8			
ECOG PS					
0-2	33	13.6	< 0.001	1	0.243
3-4	3	1.0		4.30 (0.37-49.51)	
CK					
Positive	7	18.7	0.678		
Non-positive	29	6.6			
MET IHC score (n = 33)					
1-2	10	24.8	0.013	1	0.006
3	23	5.7		2.05 (1.23-3.43)	
MET IHC distribution (n = 33)					
Focal	5	27.6	0.036	1	0.312
Diffuse	28	3.8		1.75 (0.59-5.15)	
Metastatic sites (numbers)					
1	19	18.4	0.037	1	0.378
≥2	17	2.8		1.43 (0.64-3.19)	
Initial brain metastasis					
No	25	18.4	0.036	1	0.005
Yes	11	2.8		3.86 (1.52-9.82)	
Malignant pleural effusion					
No	18	5.7	0.637		
Yes	18	12.1			
Supportive care only*					
No	29	20.1	<0.001	1	<0.001
Yes	7	1.4		11.78 (3.40-40.86)	

CI, confidence interval; CK, pancytokeratin; ECOG PS, Eastern Cooperative Oncology Group performance status; F, female; IHC, immunohistochemistry; M, male; n, number; OS, overall survival.

*Patients received only supportive care without anti-cancer therapy.

#Univariate analysis was performed using the Kaplan–Meier method and log-rank test.

^$^Multivariate analysis was performed using the backward-stepwise method for the Cox regression model.

**Figure 2:**
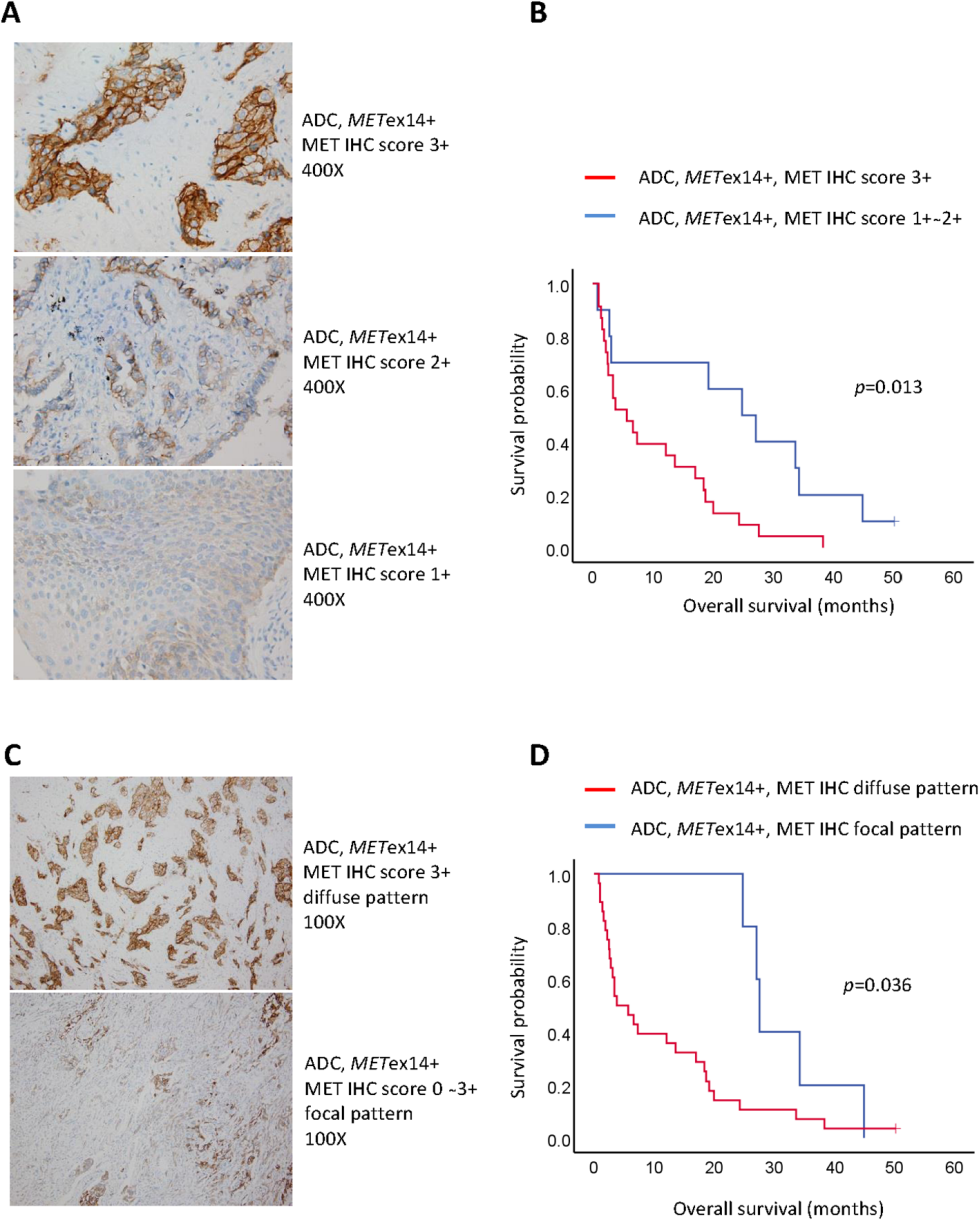
Pathological factors associated with poor prognosis in stage IV lung adenocarcinoma harboring *MET*ex14 (*MET*ex14+). **(A)** Representative figures of c-MET immunohistochemistry staining score 3+ (upper panel; original magnification: 400x), score 2+ (middle panel; original magnification: 400x), and score 1+ (lower panel; original magnification: 400x); **(B)** Kaplan-Meier curves of overall survival for score 1+ ~ 2+ and score 3+; **(C)** Representative figures of c-MET immunohistochemistry distribution patterns ― diffuse pattern (upper panel; original magnification: 100x) and focal pattern (lower panel; original magnification: 100x); **(D)** Kaplan-Meier curves of overall survival for diffuse and focal patterns.

Demographic factors, such as age (<70 vs. ≥70 years), sex, and smoking status, did not show statistically significant differences in relation to median OS (mOS). Pancytokeratin (CK) staining was not associated with differences in survival rates. Among the 33 tumors available for c-MET IHC, all *MET*ex14-positive tumors showed c-MET-positive expression and were categorized on the basis of staining scores (1+ ~ 2+ vs. 3+, **Figure 2A**). All c-MET patterns were either focal or diffuse (**Figure 2C**). We observed that patients with tumor samples showing strong (score 3+) c-MET IHC staining had a shorter mOS than those with weak or moderate (score 1+ ~ 2+) c-MET IHC staining (5.7 vs. 24.8 months, *p* = 0.013; **Figure 2B**). Similar findings were observed for the c-MET IHC distribution patterns; patients with tumor samples showing a diffuse pattern had shorter mOS than those with samples showing a focal pattern (3.8 vs. 27.6 months, *p* = 0.036; **Figure 2D**). Next, we evaluated the characteristics of the metastatic status for OS analysis. Patients with multiple initial metastatic sites (≥2) showed poorer survival outcomes than those with only one metastatic site (2.8 vs. 18.4 months, *p* = 0.037). A shorter OS was also observed in patients with metastatic brain tumors at the initial presentation (2.8 vs. 18.4 months, *p* = 0.036). The presence of malignant pleural effusion was not associated with survival outcomes. Among stage IV ADC patients with the *MET*ex14 mutation, seven patients received only supportive care without anti-cancer therapy and had a shorter mOS (1.4 months, 95% confidence interval [CI], 0.7-1.3) than those who received anti-cancer treatment (n = 29; mOS, 20.1 months; *p* < 0.001). Finally, multivariate analysis for OS revealed that a strong c-MET IHC staining score of 3+ (hazard ratio [HR]: 2.05, 95% CI: 1.23–3.43; *p* = 0.006), initial brain metastasis (HR: 3.86, 95% CI: 1.52–9.82; *p* = 0.005), and treatment with supportive care without anti-cancer therapy (HR: 11.78, 95% CI: 3.40–40.86; *p* < 0.001) were associated with poor survival outcomes (**Table 2**).

### 3.3 Univariate and multivariate analyses of prognostic factors for overall survival in stage IV adenocarcinoma patients harboring the *MET*ex14 mutation who received anti-cancer therapy

We next aimed to determine whether patient characteristics and differences in treatment modalities would affect the OS in the 29 lung ADC patients with the *MET*ex14 mutation who received at least one anti-cancer therapy (**Table 3**). All the patients had an ECOG PS score of 0-2. In univariate analysis, strong c-MET ICH staining (score 3+) was consistently associated with shorter mOS than weak-to-moderate staining (score 1+ to 2+; mOS, 7.3 and 27.1 months, respectively; *p* = 0.015). Although the c-MET IHC distribution pattern (*p* = 0.068) and initial brain metastasis (*p* = 0.061) showed a trend, the findings did not reach statistical significance. Other characteristics, such as the number of metastatic sites and malignant pleural effusion, were not associated with survival outcomes. Nevertheless, longer survival periods were observed in some subgroups. Consistently better mOS was observed in patients who received lung radiation therapy than patients who did not receive this treatment (27.6 vs. 12.1 months, *p* = 0.002). Patients who received immunotherapy showed a favorable mOS (n = 5; mOS, 44.9 months) than those who did not (n = 24; mOS, 13.6 months; *p* = 0.032). Similarly, patients treated with pemetrexed (n = 19; mOS, 20 months) showed a more favorable mOS than those who were not (n = 10; mOS, 5.7 months; *p* = 0.011). Finally, patients treated with MET TKIs (n = 6; mOS, 19.2 months) showed a trend of prolonged OS in comparison with those who did not receive MET TKI (n = 23; mOS, 13.6 months; *p* = 0.065). Other therapeutic modalities, including lung surgery, brain RT, bone or spine RT, and chemotherapy with cisplatin doublet, gemcitabine, or taxanes, did not significantly predict OS.

**Table 3:** Univariate and multivariate analyses of prognostic factors for overall survival in patients with stage IV *MET*ex14-positive adenocarcinomas who received anti-cancer treatments (n = 29).

Factor	Patients n	Univariate analysis^#^		Multivariate analysis^$^	
		Median OS (month)	*p*-value	HR (95% CI)	*p*-value
Age, years					
<70	12	18.4	0.983		
≥70	17	17.0			
Sex					
M	16	17.0	0.408		
F	13	20.0			
Smoking status					
Non-smoker	20	17.0	0.460		
Smoker/ex-smoker	9	18.4			
MET IHC score (n = 27)					
1-2	9	27.1	0.015	1	0.012
3	18	7.3		2.06 (1.17-3.62)	
MET IHC distribution (n = 27)					
Focal	5	27.6	0.068	1	0.179
Diffuse	22	7.3		2.24 (0.69-7.27)	
Metastatic sites (numbers)					
1	17	18.7	0.130		
≥2	12	6.6			
Initial brain metastasis					
No	21	19.2	0.061	1	0.005
Yes	8	3.1		5.24 (1.65-16.60)	
Malignant pleural effusion					
No	14	18.4	0.508		
Yes	15	17.0			
Lung surgical treatment*					
No	27	17.0	0.176		
Yes	2	28.4			
Lung radiotherapy					
No	19	12.1	0.002	1	0.020
Yes	10	27.6		0.26 (0.09-0.81)	
Brain radiotherapy					
No	22	18.4	0.484		
Yes	7	5.7			
Bone or spine radiotherapy					
No	21	18.4	0.605		
Yes	8	13.6			
MET inhibitor					
No	23	13.6	0.065	1	0.723
Yes	6	19.2		0.74 (0.14-4.00)	
Immunotherapy					
No	24	13.6	0.032	1	0.699
Yes	5	44.9		0.76 (0.18-3.12)	
Cisplatin doublet					
No	9	6.6	0.443		
Yes	20	18.7			
Pemetrexed					
No	10	5.7	0.011	1	0.003
Yes	19	20.0		0.20 (0.07-0.56)	
Gemcitabine					
No	19	7.3	0.309		
Yes	10	24.3			
Taxanes					
No	16	6.6	0.284		
Yes	13	24.3			

CI, confidence interval; F, female; IHC immunohistochemistry; M, male; n, number; OS, overall survival; RT, radiotherapy.

* One patient received wedge resection for surgical biopsy. Another patient initially received right upper lobectomy but concurrent malignant pleural effusion was found during operation.

# Univariate analysis was performed using the Kaplan–Meier method and log-rank test.

^$^A multivariate analysis was performed using the backward-stepwise method for the Cox regression model.

Multivariate analyses for 29 stage IV ADC patients carrying the *MET*ex14 mutation were performed, and the variables with *p*-values less than 0.1 in the univariate analysis were included (**Table 3**). After adjusting for clinicopathological factors, a significantly longer OS was observed in patients who received pemetrexed (HR: 0.20; 95% CI: 0.07–0.56; *p* = 0.003) and those who were treated with lung radiotherapy (HR, 0.26; 95% CI: 0.09–0.81; *p* = 0.020). Similar to the findings for all stage IV ADC patients harboring the *MET*ex14 mutation, anti-cancer therapy with initial brain metastasis (HR: 5.24, 95% CI: 1.65–16.60; *p* = 0.005) and strong c-MET IHC staining (HR: 2.06, 95% CI: 1.17–3.62; *p* = 0.012) consistently predicted poor survival outcomes in these 29 patients.

### 3.4 Survival outcomes of stage IV *MET*ex14-positive lung cancer patients in comparison with those without the *MET*ex14 mutation

**Figure 3:**
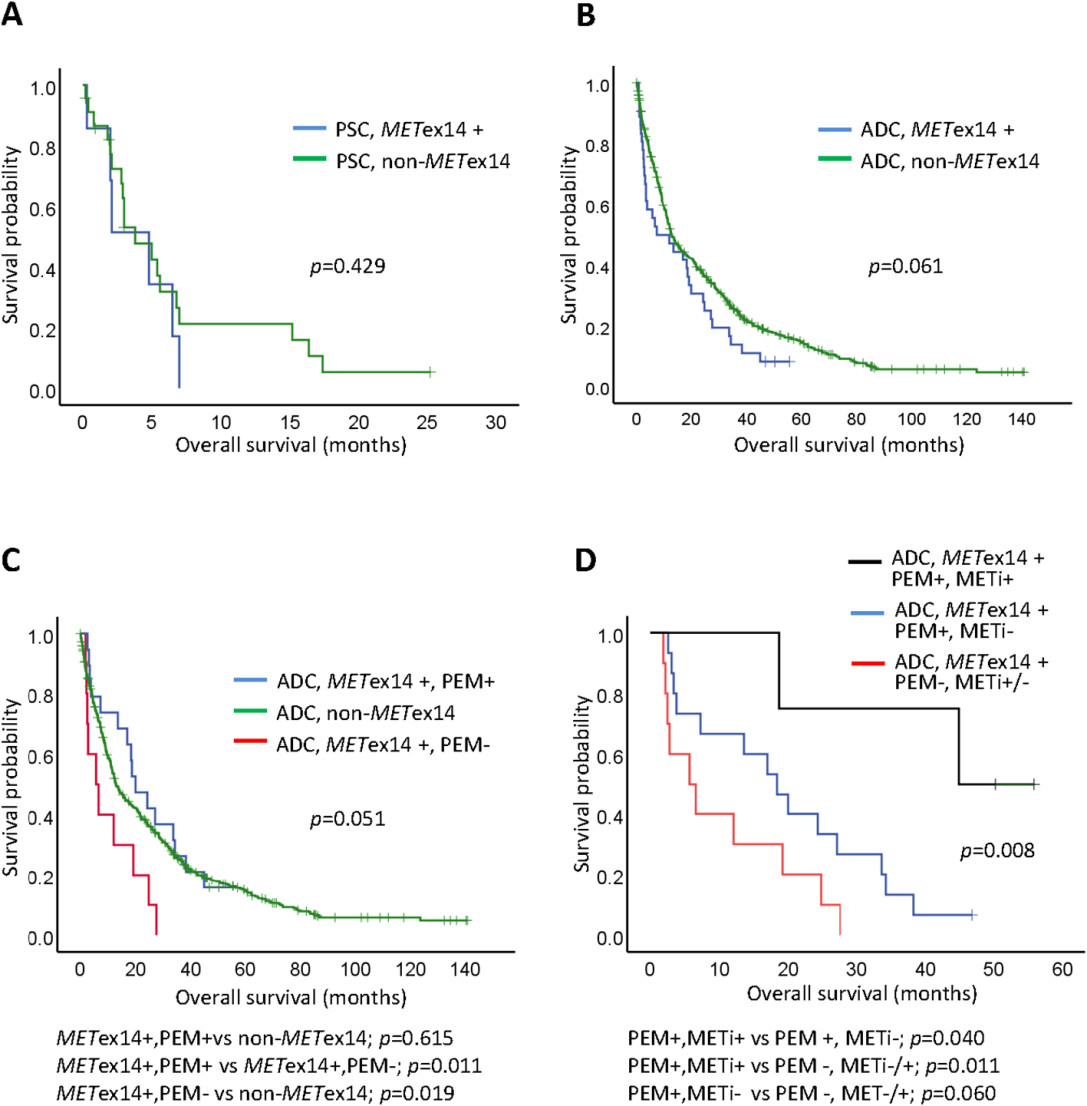
Kaplan-Meier curves of overall survival (OS) for Stage IV lung cancers. **(A)** Pulmonary sarcomatoid carcinoma (PSC) in *MET*ex14 positive (*MET*ex14+) or non-*MET*ex14 patients; **(B)** Adenocarcinoma (ADC) patients with *MET*ex14 (*MET*ex14+) or non-*MET*ex14 patients; **(C)** ADC patients with *MET*ex14 who received pemetrexed (PEM+), ADC patients with *MET*ex14 who did not received pemetrexed (PEM-), and ADC with non-*MET*ex14 patients; pairwise comparisons for *p*-value were shown below the panel; **(D)** ADC patients with *MET*ex14 who received sequential pemetrexed (PEM+) and MET inhibitor (METi+), who received pemetrexed (PEM+) without MET inhibitor (METi-), and who did not receive pemetrexed (PEM-) and with or without MET inhibitor (METi+/-); pairwise comparisons for *p*-value were shown below the panel.

For the stage IV PSC cases in our cohort, the estimated mOS was 4.8 months in the seven *MET*ex14-positive patients and 3.8 months in the 23 patients without *MET*ex14, indicating a similar mOS and poor survival in both groups (*p* = 0.429; **Figure 3A**). Among the *MET*ex14-positive patients, five of the seven PSC patients were poor chemotherapy responders. Most of these patients received less than four courses of first and/or second-line cisplatin doublet-based chemotherapy with rapid progression. One patient with an ECOG PS of 4 died within 2 weeks of diagnosis who received best supportive care. Another patient received a course of pembrolizumab and was lost to follow-up. After excluding patients who received only supportive care, similar mOS was observed between *MET*ex14-positive patients (n=6; mOS, 4.8 months) and non-*MET*ex14 patients (n=18; mOS, 5.4 months, *p*=0.388; **Supplementary Figure 1A**).

We next evaluated and compared the survival outcomes of patients with stage IV lung ADC with and without the *MET*ex14 mutation. The mOS was 7.3 months in *MET*ex14-positive patients (n = 36; 95% CI: 0-18.9) and 12.9 months in the patients without the *MET*ex14 mutation (n = 524; 95% CI: 10.8-15.0; **Figure 3B**). Although the OS was shorter in patients with the *MET*ex14 mutation, this trend did not show statistical significance (*p* = 0.061). After excluding patients who received only supportive care, a comparable survival outcome was observed between *MET*ex14-positive patients (n=29; mOS, 18.4 months; 95% CI: 9.4-27.4) and non-*MET*ex14 patients (n=469; mOS, 15.9 months; 95% CI: 12.3-19.5 *p*=0.236; **Supplementary Figure 1B**).

We further evaluated 29 patients who had been treated with pemetrexed and/or MET TKI. The detailed duration of whole treatment regimens for patients with *MET*ex14 were shown in **Supplementary Figure 2**. Pairwise comparisons of the 524 patients without the *MET*ex14 mutation (non-*MET*ex14), 19 *MET*ex14-positive patients receiving pemetrexed (*MET*ex14+, PEM+), and 10 *MET*ex14-positive patients treated with chemotherapeutic agents other than pemetrexed (*MET*ex14+, PEM-) were performed (**Figure 3C**). As mentioned in the univariate analysis, *MET*ex14+, PEM+ patients showed better mOS than *MET*ex14+, PEM- patients (*p* = 0.011). No significant difference in mOS was observed between *MET*ex14+, PEM+ patients (20.0 months) and non-*MET*ex14 patients (*p* = 0.615). However, *MET*ex14+, PEM- ADC patients had a worse mOS (5.7 months) than the non-*MET*ex14 patients (*p* = 0.019; **Figure 3C**). Six patients received one or two lines of MET TKIs (**Supplementary Table 2**). Four patients treated with sequential pemetrexed with MET TKIs (at different time periods) had an mOS of NR (not reached), which was longer than that of the 15 patients who received pemetrexed without MET TKIs (mOS, 18.4 months; *p* = 0.040; **Figure 3D**), and was much better than that of the 10 patients who did not receive pemetrexed (including two patients who received MET TKIs but no pemetrexed), whose mOS was 5.7 months (*p* = 0.011; **Figure 3D**).

## 4 Discussion

Several clinical studies and trials have reported that NSCLC patients harboring *MET*ex14-positive tumors benefit from MET TKIs (9, 11, 13, 17, 21). However, not all patients showed clinical efficacy, and the response duration was limited. In the real world, some patients do not receive a specific MEK-TKI. In this study, we performed a multi-faceted evaluation of several prognostic factors associated with survival outcomes in a cohort of patients with lung cancer. We first successfully performed RNA-based PCR analysis and identified higher frequencies of *MET*ex14 in PSC, followed by ADC, and smaller frequencies in other lung cancer subtypes. For patients with stage IV lung ADC, we comprehensively analyzed the potential variables influencing survival outcomes. We showed that initial brain metastases and strong MET IHC staining may help predict OS. These results provide important information and shed light on the survival characteristics of lung cancer patients with *MET*ex14-positive tumors.

Previous studies reported that the overall incidence of the *MET*ex14 mutation was approximately 20%-30% in PSC and 3%-4% in ADC (6, 22). Our study reported a similar frequency of the *MET*ext14 mutation in PSC. Although pleomorphic carcinoma is categorized as a subtype of PSC in the 2015 World Health Organization (WHO) classification of lung tumors (23), we classified it as an independent subtype of lung cancer because the frequency of the *MET*ex14 mutation in pleomorphic carcinoma (5%) was quite different from that in PSC (24%). Moreover, the *MET*ex14 mutation was detected in 6.6% of lung ADC patients without *EGFR* and *ALK* mutations. Other characteristics of *MET*ex14-positive lung cancer, such as a predominance in female patients and an association with smoking, have been reported in previous studies (22, 24) but were not shown in our cohort and other studies (25, 26). The advanced age of patients with the *MET*ex14 mutation has been reported in the current and previous studies (21, 22, 25). Finally, these patients were more fragile and generally had a poorer ECOG PS than those without the *MET*ex14 mutation. As reported in previous studies, these demographic characteristics were associated with a poor OS, which may contribute to a highly aggressive subtype and short survival outcome for lung cancer patients carrying *MET*ex14-positive tumors (8, 27).

PSC is considered an aggressive subtype of lung cancer. Patients with PSCs generally show rapid progression, early metastasis, and dismal prognosis (28). The mOS of stage IV PSC patients was only 5.4 months in results from the National Cancer Database (29) and 2 months in those from the Surveillance, Epidemiology, and End Results (SEER) database (30). Patients who received anti-cancer chemotherapy still had a short mOS of only 6 months (31). A comparable and dismal survival outcome was observed in the current study; the mOS was <5 months for the advanced-stage patients with *MET*ex14-positive and *MET*ex14-negative PSC. None of the patients with *MET*ex14-positive PSC received targeted therapy, and most were not chemotherapy responders. This observation was consistent with the findings of a previous study that described the characteristics of chemoresistance and early progression in PSC (32). At present, evidence of treatment efficacy for *MET*ex14-positive PSC is inconclusive, and immune checkpoint inhibitors (ICIs) have been reported to show good efficacy in limited cases (33, 34). However, a pooled analysis of published data demonstrated that the *MET*ex14 mutation was not associated with tumor response (35). The impact of MET TKIs on OS in advanced PSC is also still unclear and variable since most reports either had small patient populations or were case reports, and described patients treated with different MET inhibitors (15, 17, 36). Lu et al. recently reported that 25 stage III or IV PSC patients with the *MET*ex14 mutation who were treated with savolitinib, a selective MET TKI, showed promising results with a response rate of 40% and a median PFS of 5.5 months (35), providing a beacon of hope for such dismal cases.

We identified several potential prognostic factors that predicted the OS in stage IV ADC patients harboring *MET*ex14 mutations. Pathological factors, including the staining score and the distribution of c-MET IHC staining, may help predict the OS. In particular, strong c-MET IHC staining with a score of 3+ was consistently associated with a short OS in both univariate and multivariate analyses for patients who received anti-cancer therapy. Awad et al. had previously reported that c-MET IHC staining in stage IV *MET*ex14-positive NSCLC was significantly stronger than that in stage I to III NSCLC with the *MET*ex14 mutation (27). The observation that strong MET expression could predict shorter OS in NSCLC (37, 38) suggests that a high MET IHC staining score may be an indicator of aggressive behavior in *MET*ex14-positive ADC (16, 39).

Among several treatment modalities, lung radiotherapy was associated with a longer mOS in both univariate and multivariate analyses. Treatment of primary lung tumors with thoracic radiotherapy has been reported to effectively ameliorate clinical respiratory symptoms, reduce tumor size, control recurrence, and prolong survival in patients with advanced NSCLC (40, 41). In our study, among 10 patients treated with lung radiotherapy, 9 patients received lung and/or mediastinal RT and other systemic therapies (chemotherapy or MET TKI) at different times and showed an mOS of 33.7 months (95% CI, 15.9-51.5), which was significantly longer than that in patients who received systemic therapy alone (mOS, 12.1 months; 95% CI, 2.1-22.0). Although MET activation plays an important role in conferring resistance to ionizing radiation by altering intracellular DNA damage response pathways in various cancer types (42), the underlying biological mechanism for prolonged OS in *MET*ex14-positive lung ADC patients receiving a combination of systemic therapy and local RT remains unclear. Radiotherapy may have diminished the resistance to systemic therapy and chemotherapy or TKI treatment may have enhanced radiosensitivity, thereby prolonging the treatment period and improving survival outcomes (43).

In multivariate analysis, initial brain metastasis was an independent risk factor for poor survival outcome in stage IV lung ADC patients harboring the *MET*ex14 mutation. The frequency of initial brain metastasis in this patient population was 30% (11 of 36), and the median OS was only 2.8 months for all 11 patients and 3.1 months for the eight patients who received anti-cancer therapy. Among them, two patients received crizotinib and six patients received brain RT plus standard chemotherapy. This mOS was inferior to that described in a previous report, which demonstrated a 6-month median OS in NSCLC with brain metastasis at initial presentation (44). The short OS in our cohort may be associated with the lack of effective treatment in most cases. Currently, the highly selective and potent MET inhibitors capmatinib and tepotinib are recommended and approved for the treatment of such patients because of their ability to cross the blood–brain barrier. A rapid partial response and impressive duration of response were reported with these inhibitors in patients with the *MET*ex14 mutation (45), and further follow-up data may be necessary to determine whether campatinib or tepotinib can prolong the OS in such patients.

Pemetrexed treatment is another strong predictor of OS for stage IV ADC patients with the *MET*ex14 mutation, which may be partly explained as a small population with MET TKI therapy in this study. However, pemetrexed–carboplatin plus pemetrexed maintenance regimen is currently used as initial chemotherapy for advanced NSCLC without targetable driver mutations (46), and may play a role in the treatment of advanced-stage ADC patients with the *MET*ex14 mutation when they are not able to receive specific MET TKIs or after MET TKI failure. Other chemotherapeutic agents, such as gemcitabine and taxanes, however, were not associated with favorable survival outcomes. Although *MET*ex14-positive ADC patients were shown to respond to platinum doublet therapy with a disease control rate of 80% in a study with small case number (47), we suggest that pemetrexed-based chemotherapy should be considered first if these patients need chemotherapy (48). Further prospective studies are needed to evaluate the role of pemetrexed in patients with advanced ADC with the *MET*ex14 mutation.

The present study had several limitations. Patient recruitment and data collection were retrospective, resulting in an inherent selection bias. Moreover, because of the relative rarity of *MET*ex14 in lung cancer, the imbalance in the number of mutation-positive and mutation-negative patients limited the viability of the analyses. In addition, intrinsic analysis of OS for stage IV ADC disease with the *MET*ex14 mutation may have been affected by the limited number of cases in each group. Finally, the type, combination, and sequence of chemotherapy, MET TKI, and immunotherapy varied among the study patients. Nevertheless, the present study provided crucial insights into the characteristics, associated factors, and survival outcomes of lung cancer patients with the *MET*ex14 mutation. Further large-scale prospective studies focusing on these prognostic factors are necessary to overcome these limitations.

## 5 Conclusion

In both lung ADC and PSC, patients with and without the *MET*ex14 mutation showed comparable survival outcomes. A higher frequency of *METex14* mutations was detected in PSC patients and these patients had poor overall survival. In lung ADC, pemetrexed-based chemotherapy (with or without MET TKI), strong c-MET ICH staining, brain metastasis at initial presentation, and lung radiotherapy were independent prognostic factors associated with survival outcomes. These findings provide information that can be expected to be important in clinical settings.

## Data availability statement

The original contributions presented in the study are included in the article/**Supplementary Material**. Further inquiries can be directed to the corresponding author.

## Ethics statement

The studies involving human participants were reviewed and approved by The study was conducted according to the guidelines of the Declaration of Helsinki and approved by the Institutional Review Board of the National Taiwan University Hospital, Taipei, Taiwan (201509070RINA and 201103013RC). The patients/participants provided their written informed consent to participate in this study.

## Author contributions

C-HG and J-YS designed the study and analyzed the data. M-SH contributed to pathological analysis. Y-LC and Y-NL performed genetic mutation analysis. C-HG wrote the manuscript. C-HG, M-SH, Y-NL, S-GW, and J-YS edited the manuscript. All authors contributed to the article and approved the submitted version.
