# Supplementary material for: Survival outcomes and prognostic factors of lung cancer patients with the MET exon 14 skipping mutation: A single-center real-world study
Source: Front Oncol. 2023 Mar 9;13:1113696. doi: 10.3389/fonc.2023.1113696 (PMC10034335; doi:10.3389/fonc.2023.1113696)

Supplementary Material

Survival outcomes and prognostic factors of lung cancer patients with the MET exon 14 skipping mutation: A single-center real-world study

Chien-Hung Gow, Min-Shu Hsieh, Yi-Lin Chen, Yi-Nan Liu, Shang-Gin Wu and Jin-Yuan Shih*

*** Correspondence:** Jin-Yuan Shih: jyshih@ntu.edu.tw

**Supplementary Table 1**. Frequency and characteristics of *MET*ex*14* mutations in different lung cancer types

| **Characteristics** | **Adenocarcinoma**  **(n = 53)** | **Sarcomatoid**  **(n = 9)** | **Squamous cell carcinoma (n = 4)** | **Small cell lung cancer (n = 1)** | **Pleomorphic carcinoma (n = 1)** | **NSCLC**  **-NOS (n = 1)** |
| --- | --- | --- | --- | --- | --- | --- |
| *MET*ex14 Frequency | 53/803 (6.6%) | 9/37 (24.3%) | 4/107 (3.7%) | 1/159 (0.6%) | 1/19 (5%) | 1/80 (1.3%) |
| Age: <70/≥70 | 19/34 | 4/5 | 2/2 | 1/0 | 0/1 | 0/1 |
| Sex: Male/Female | 34/19 | 6/3 | 2/2 | 1/0 | 1/0 | 0/1 |
| Smoking: Yes/No | 21/32 | 3/6 | 2/2 | 1/0 | 1/0 | 0/1 |
| Stage: I/II/III/IV | 10/1/6/36 | 1/1/0/7 | 0/1/0/3 | 0/0/0/1 | 0/0/0/1 | 0/0/0/1 |

Abbreviations: NSCLC-NOS, non-small-cell lung cancer-not otherwise specified.

**Supplementary table 2.** Stage IV lung ADC patients with the *MET*ex14 mutation who had received one or at least two lines of MET inhibitors (n = 6).

| **Case No.** | **MET TKIs (months)** | **Side effect*** | **Line** | **Response^#^** | **Alive/Dead** |
| --- | --- | --- | --- | --- | --- |
| 2 | Crizotinib (12.5)  Campatinib (31~) | Leukopenia | 2^nd^  3^rd^ | SD  PR | Alive |
| 13 | Crizotinib (19.3) |  | 1^st^ | SD | Dead |
| 1 | Tepotinib (14.8) |  | 2^nd^ | PR | Alive |
| 4 | Crizotinib (2)  Campatinib (7.2) | Hepatitis | 4^th^  5^th^ | SD  SD | Dead |
| 14 | Campatinib (8.8) |  | 2^nd^ | SD | Dead |
| 25 | Crizotinib (1.5) | Interstitial pneumonitis | 2^nd^ | PD | Dead |

Abbreviations: ADC, adenocarcinoma; PD, progressive disease; PR, partial response; SD, stable disease; TKI, tyrosine kinase inhibitor.

* The side effects indicated that severe disease led to discontinuation of the MET inhibitor.

#The Response Evaluation Criteria in Solid Tumors Group (RECIST, version 1.0) was used to evaluate the best overall response.

**Supplementary figure 1. Kaplan-Meier curves of overall survival (OS) for Stage IV lung cancers who received anti-cancer therapy. (A) Pulmonary sarcomatoid carcinoma (PSC) in *MET*ex14 positive (*MET*ex14+) or non-*MET*ex14 patients; (B) Adenocarcinoma (ADC) patients with *MET*ex14 (METex14+) or non-*MET*ex14 patients.**

**
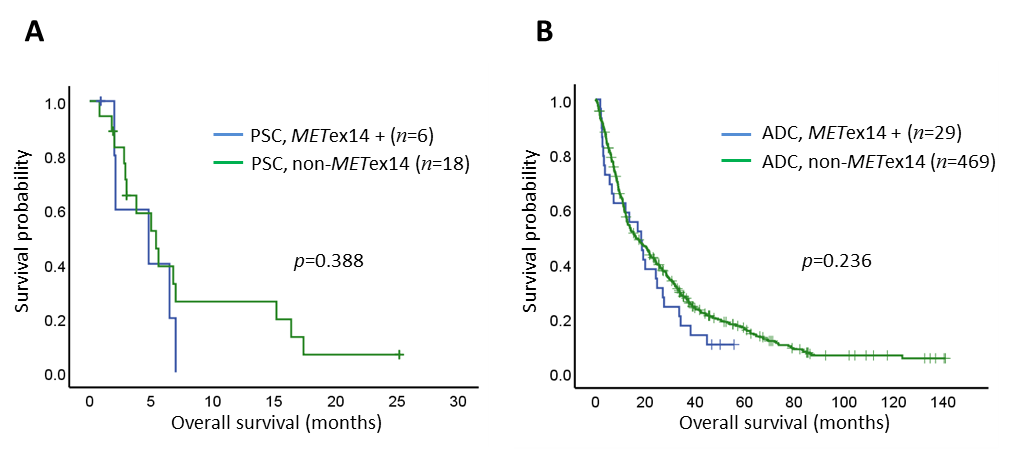
**

**Supplementary figure 2. The duration of treatments with chemotherapy, targeted and non-targeted therapies, or immunotherapy for stage IV patients with *MET*ex14-mutant lung ADC.**


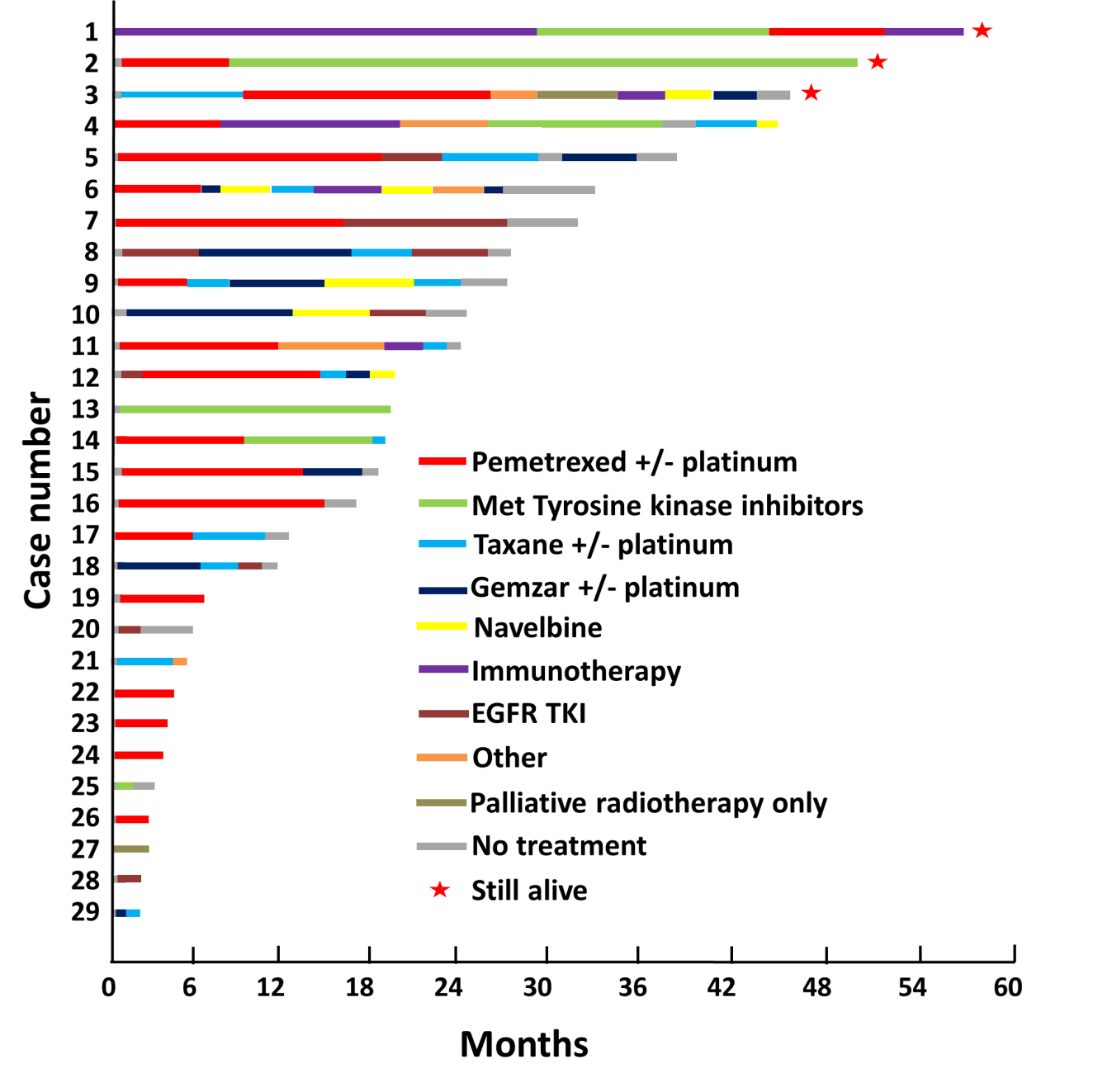

Supplement: Supplementary file 1 [file DataSheet_1.docx]
